# Compound Fracture of Inferior Maxillary—Treatment of, Etc.

**Published:** 1892-09

**Authors:** T. D. McKown

**Affiliations:** Chicamauga, Ga.


### COMPOUND FRACTURE INFERIOR MAXILLARY—TREATMENT OF, ETC.

BY T. D. MCKOWN, M. D., CHICAMAUGA, GA.

The case that I will give you, is a man who, while driving a four-horse wagon, heavily loaded with trestle timber, fell off the wagon and was run over by the rear wheels. The load, I suppose, was easily 3,000 pounds, and the accident occurred while going down a steep rocky hill. I give these details in order that you may imagine the magnitude of the injuries sustained. The wheel passed over the right side of the head and face, crushing the left side into the rocks, it then passed over the left clavicle and left forearm. I was summoned at once and found following injuries:

Compound fracture superior maxillary, compound comminuted fracture inferior maxillary, fracture clavicle and first four ribs, compound fracture bones forearm. These fractures all occurred on the same side, which made them (to me) more difficult to treat. I will give the treatment of the fractures of maxillary bones in as few words as possible.

I cleansed the wounds and removed every particle of gravel, sand, etc. I then dressed the wounds antiseptically, and placed the broken bones in position, put on ordinary pasteboard splint and comprised it with the four-tail bandage. But the next day the broken ribs had made some injury to the lung, and my patient had a well marked case of circumscribed pneumonia with delirium. He would tear the bandages off and get the broken bones in worse shape than they were before they were placed in position. I could not rely on the bandages of pasteboard for two reasons: First, I could not keep it in position; second, it interfered with the subsequent dressing of the wounds, for every time the splint is removed, the boxes will get "out of kilter," unless an assistant will hold the jaw fast, which entails an immense deal of suffering to the patient. To overcome those difficulties, I put a tight fitting plaster Paris splint that enveloped the whole jaw. This can be done very easily and effectually. I used a two-inch bandage, commencing at a point immediately under the chin, carrying the bandage around the top of the head (just missing the ear) and around the chin on opposite side. It should be carried around several times, and then commencing at syphysis menti, carrying bandage around nape of the neck and chin several times, then carrying the bandage up to a point just above the ear, running it around the occiput and forehead several times, fixing the splint firmly. I cut windows sufficiently large to allow drainage and dressing of external wounds. Suffice it to say, that patient did not pull this splint off. It has now been thirty days since he was hurt, and I am sure that the man will get well with little disfiguration.

Up to the present, I have extracted two very small pieces of bone, and believe there will be a number of them to come out before patient has complete union. It is possible that several months will have elapsed before he can use the jaw, but I believe I will get good union and a good jaw.

I shall use the plaster Paris splint in my next case, believing it to be the most rational way to treat all fractures of the superior maxillary. I am indebted to Dr. D. G. Elder for valuable assistance in fitting the splint.

